# Slit2–Robo4 signal pathway and tight junction in intestine mediate LPS-induced inflammation in mice

**DOI:** 10.1186/s40001-024-01894-5

**Published:** 2024-06-27

**Authors:** Lv Wang, Yingtai Chen, Hao Wu, He-hua Yu, Linhao Ma

**Affiliations:** 1 Department of Emergency and Critical Care Medicine, Shanghai Changzheng Hospital, Naval Medical University, Shanghai, 200003 People’s Republic of China; 2 Emergency Department, Baoshan Branch of Renji Hospital, School of Medicine, Shanghai Jiaotong University, Shanghai, 200444 People’s Republic of China

**Keywords:** Sepsis, Intestine, Inflammation, Slit2–Robo4, NLRP3 inflammasome, Tight junction

## Abstract

**Background:**

Sepsis is one of the most common clinical diseases, which is characterized by a serious and uncontrollable inflammatory response. LPS-induced inflammation is a critical pathological event in sepsis, but the underlying mechanism has not yet been fully elucidated.

**Methods:**

The animal model was established for two batches. In the first batch of experiments, Adult C57BL/6J mice were randomly divided into control group and LPS (5 mg/kg, i.p.)group . In the second batch of experiments, mice were randomly divided into control group, LPS group, and LPS+VX765(10 mg/kg, i.p., an inhibitor of NLRP3 inflammasome) group. After 24 hours, mice were anesthetized with isoflurane, blood and intestinal tissue were collected for tissue immunohistochemistry, Western blot analysis and ELISA assays.

**Results:**

The C57BL/6J mice injected with LPS for twenty-four hours could exhibit severe inflammatory reaction including an increased IL-1β, IL-18 in serum and activation of NLRP3 inflammasome in intestine. The injection of VX765 could reverse these effects induced by LPS. These results indicated that the increased level of IL-1β and IL-18 in serum induced by LPS is related to the increased intestinal permeability and activation of NLRP3 inflammasome. In the second batch of experiments, results of western blot and immunohistochemistry showed that Slit2 and Robo4 were significant decreased in intestine of LPS group, while the expression of VEGF was significant increased. Meanwhile, the protein level of tight junction protein ZO-1, occludin, and claudin-5 were significantly lower than in control group, which could also be reversed by VX765 injection.

**Conclusions:**

In this study, we revealed that Slit2-Robo4 signaling pathway and tight junction in intestine may be involved in LPS-induced inflammation in mice, which may account for the molecular mechanism of sepsis.

## Introduction

Sepsis is one of the most common clinical diseases in the emergency department and ICU, which is characterized by the high expression of various inflammatory mediators and cytokines caused by the invasion of pathogenic microorganisms into the human body [1–3]. Epidemiologic studies and clinical investigations have shown that lipopolysaccharide (LPS), the main component of cell wall of Gram-negative bacteria, is an important cause of sepsis induced by bacterial infection [4, 5]. Many medical documents showed that LPS can induce the synthesis of a large number of pro-inflammatory cytokines such as interleukin-1β (IL-1β), interleukin-18 (IL-18), tumor necrosis factor-α (TNF-α), and interferon-γ (IFN-γ). The excessive secretion of these cytokines, in turn, can further induce more serious and unregulated inflammatory reactions [6–8]. Although there are more and more experimental and clinical studies on sepsis, the pathogenesis of sepsis and its underlying molecular mechanism have not yet been fully elucidated up to now.

Studies have shown that inflammasomes are involved in the pathophysiological process of various inflammatory diseases [9–11]. At present, the most clearly studied inflammasome is nucleotide-binding oligomerization domain (NOD)‑like receptor protein 3 (NLRP3) inflammasome, which is composed of NLRP3, apoptosis‑associated speck‑like protein (ASC) and aspartate proteolysis. Aspartate proteolysis is a protein complex composed of cysteinyl aspartate‑specific proteases‑1(caspase‑1) protein which can cut pro‑interleukin1β (pro-IL-1β) and pro‑interleukin 18 (pro-IL-1β) into active IL‑1β and IL‑18 [12]. NLRP3 inflammasome complex can be activated by a broad spectrum of stimuli, including bacteria, virus, fungi, and components of dying cells [13–15]. The activation of NLRP3 inflammasome can produce a large number of inflammatory factors, which can influence the permeability of endothelial cells [16].

More and more evidence has indicated that the dynamic balance of tight junction proteins in endothelial cells is related to a variety of inflammatory mediators and innate immunity, even can coordinate the host response in sepsis [17, 18]. The barrier properties of vascular and intestinal endothelial cells are essential for maintaining homeostasis of the internal environment, impairment of which is closely related to the occurrence and/or progression of various diseases, including enteritis, ulcerative colitis, stroke, cancer, sepsis, and neurodegenerative diseases [19, 20]. It is well known that these barrier properties are related to the aggregation of various proteins on endothelial cells to form specific membrane domains, such as adhesion junctions, gap junctions, and tight junctions [21]. Tight junctions are composed of different proteins such as occludin, claudin family members and zonula occludins (ZO) family members, which form a selective molecular channel [22]. This channel usually only allows small ions and uncharged molecules to pass through, and the change of its permeability is often related to the occurrence of diseases.

Slit Homolog (Slit) and Roundabout Homolog (Robo) are evolutionarily conserved proteins, which are widely expressed in different tissues such as heart, lung, and intestine [23, 24]. Slit can be divided into three subtypes, Slit1–3, and which has four membrane receptors, Robo1–4. Recently, more and more literature indicated that secretory protein Slit2 and its receptor Robo4 play an important role in regulating the fluidity and permeability of endothelial cells [25, 26]. For instance, Slit2 bind to the receptors Robo1 and Robo4, and then induces a series of intracellular signaling events. Slit2 regulates angiogenesis and protects endothelial integrity during sepsis and HIV infection [27]. However, the regulation of Slit2–Robo4 signal pathway in inflammatory process is still unclear. Therefore, it is urgent to understand the role of LPS in regulating Slit2 and Robo4 expression and disease progression.

The aim of this study is to determine the underlying mechanism of LPS-induced inflammation in sepsis. This study was designed to test the hypothesis that Slit2–Robo4 signaling pathway and tight junction in intestine may be involved in LPS-induced inflammation in mice model of sepsis.

## Material and methods

### Animals

Adult C57BL/6 J male mice (weighing 18 ± 1 g) were purchased from Shanghai Laboratory Animal Co., Ltd (Shanghai, China) and housed in a standard SPF rodent laboratory with a 12:12 h light:dark cycle. The environmental parameters of all animals were as follows: controlled temperature (22 ± 2 °C), humidity (50 ± 10%), standard diet and water ad libitum.

### Animal model of LPS-induced sepsis

The animal model was established for two batches. In the first batch of experiments, 16 mice were randomly divided into control (Con) group and LPS group (*n* = 8 in each group). The LPS group was intraperitoneally injected with 0.1 ml LPS solution (solvent: PBS, 5 mg/kg, i.p.), and the control (Con) group was injected with the same amount of 0.9% saline. After 24 h, mice were anesthetized with isoflurane, blood and intestinal tissue were collected for further study. In the second batch of experiments, 24 mice were randomly divided into control (Con) group, LPS group, and LPS + VX765 group (*n* = 8 in each group). The LPS group was intraperitoneally injected with 0.1 ml LPS solution (solvent: PBS, 5 mg/kg, i.p.), LPS + VX765 group were injected with 0.1 ml VX765 (solvent: DMSO, 10 mg/kg, i.p.) at the same time of LPS injection, and the control (Con) group was injected with the same amount of saline. All drug concentrations used in this study were confirmed based on literature and preliminary trials [28, 29]. After 24 h, mice were anesthetized with isoflurane, blood and intestinal tissue were collected for further study.

### Tissue immunohistochemistry

The intestinal tissues in each group were cut and washed by cold PBS solution (4 ℃). All intestine first fixed with 10% formalin and then embedded in paraffin wax, for cutting into 3-mm sections. Deparaffinized tissue sections were stained with Slit2, Robo4, and VEGF antibodies (Abcam, CA, USA) at 1:200 and then observed under a light microscope.

### Western blot analysis

Total protein was isolated from the intestine by homogenization in cell lysis buffer (Sangon Biotech Co., Ltd., Shanghai, China), and then centrifuged at 12,000 × *g* for 30 min at 4 ℃. The proteins (5 μg/μL) were separated by 10% SDS-PAGE in Tris–glycine–SDS buffer. Separated proteins were transferred to NC membranes which were blocked with 5% skim milk (Bright Dairy Co., Ltd, China) for one hour at room temperature and then incubated with a 1:500 dilution of the individual primary antibodies anti-Slit2 (ab134166), anti-Robo4 (ab180824), anti-VEGF (ab150375), anti-NLRP3 (ab263899), anti-ASC (ab2236), anti-Caspase1 (ab207802), anti-ZO1 (ab61357), anti-Claudin5 (ab131259), anti-Occludin (ab167161), and anti-β-actin (ab6276) (Abcam, CA, USA). The antibodies were diluted in 3% BSA in Tris-buffered saline containing 0.1% Tween 20 (TBST), and applied overnight at 4℃. Membranes were washed and incubated with HRP-conjugated secondary antibodies (Proteintech, USA). Protein bands were detected using the Western Blot Luminol Reagent (Bio-rad, USA).

### ELISA assays of inflammatory cytokines

Firstly, blood samples were centrifuged at 1000 × *g* for 5 min, and then serum was collected for further study. Next, IL-1β and IL-18 were determined by ELISA kits according to the instructions of the product (R&D, USA).

### Statistical analysis

The data were analyzed using one-way ANOVA followed by the Dunnett's multiple range test using SPSS 21.0. All data are expressed as means ± SEM, and in all group, differences were considered statistically significant at *p* < 0.05.

## Results

### LPS can induce inflammatory response mediated by NLRP3 inflammasome

**Fig. 1:**
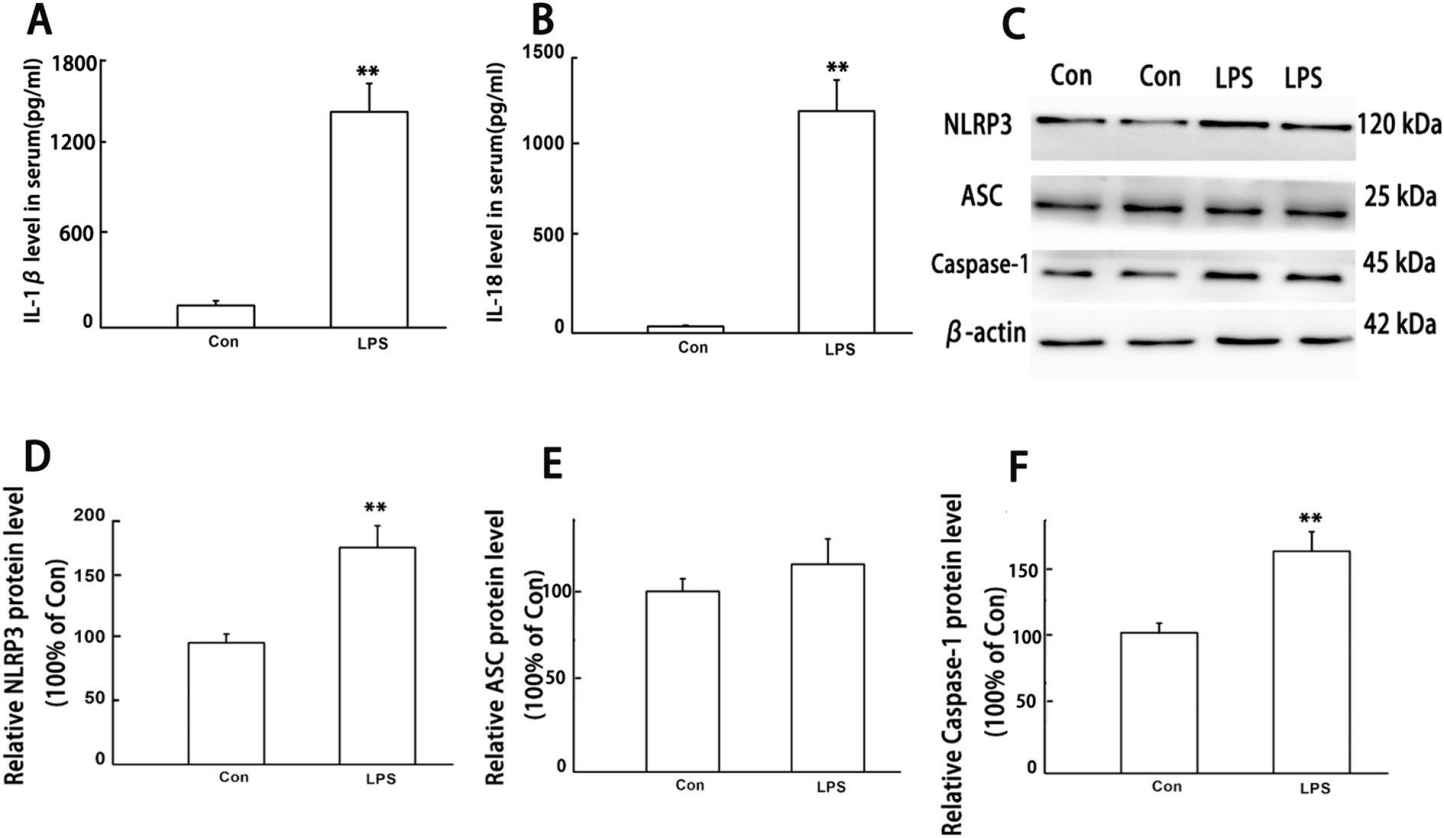
LPS-induced inflammation in serum and activation of NLRP3 inflammasome in intestine. IL-1β and IL-18 were detected by ELISA, NLRP3 inflammasome were detected by WB. All experiments were repeated twice. **A** IL-1β, **B** IL-18, **C** representative protein bands, **D** protein expression levels of NLRP3; **E** protein expression levels of ASC, **F** protein expression levels of Caspase-1. Data are presented as the mean ± SEM, *n* = 8 in each group. ^**^P < 0.01 vs. control. *Con* control

Firstly, in order to investigate the effects of LPS on C57BL/6 J mice, the LPS group mice were injected with LPS solution (5 mg/kg, i.p.), and the control (Con) group mice were injected with the same amount of saline. After 24 h, serum and intestinal tissue were collected for evaluating the expression of IL-1β, IL-18 and NLRP3 inflammasome by ELISA and western blot. ELISA results showed that LPS exposure for 24 h can induce significant high levels of IL-1β and IL-18 in serum when compared to Con group (Fig. 1A, B). Western blot results showed that the expression of NLRP3 and Caspase-1 protein increased significantly (Fig. 1C–F).

### LPS can increase the permeability of intestine and activation of Slit2–Robo4 signal pathway

**Fig. 2:**
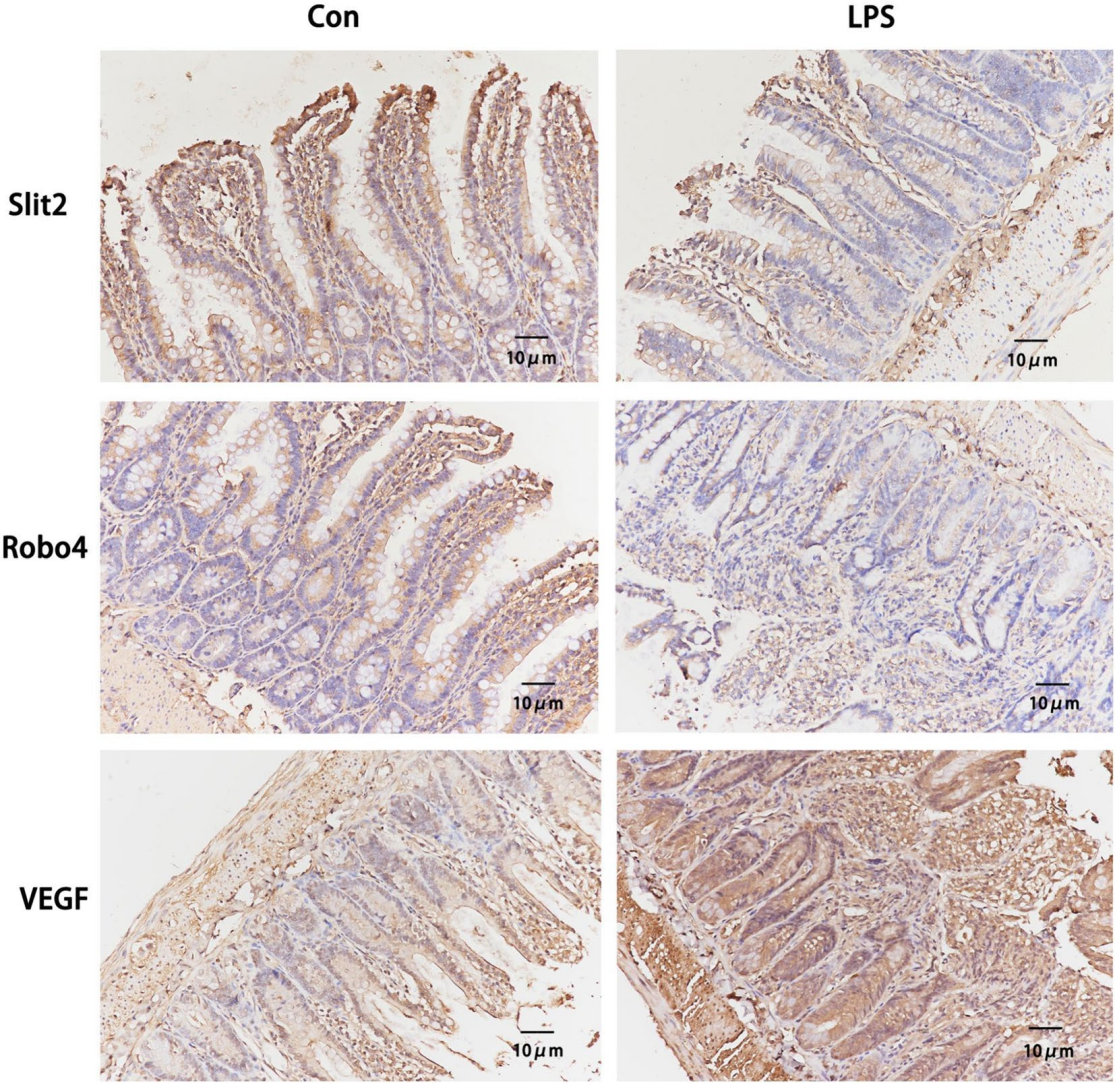
LPS-induced inhibition of Slit2–Robo4 signal pathway and activation of VEGF. Slit2, Robo4 and VEGF were detected by immunohistochemistry. All experiments were repeated twice

**Fig. 3:**
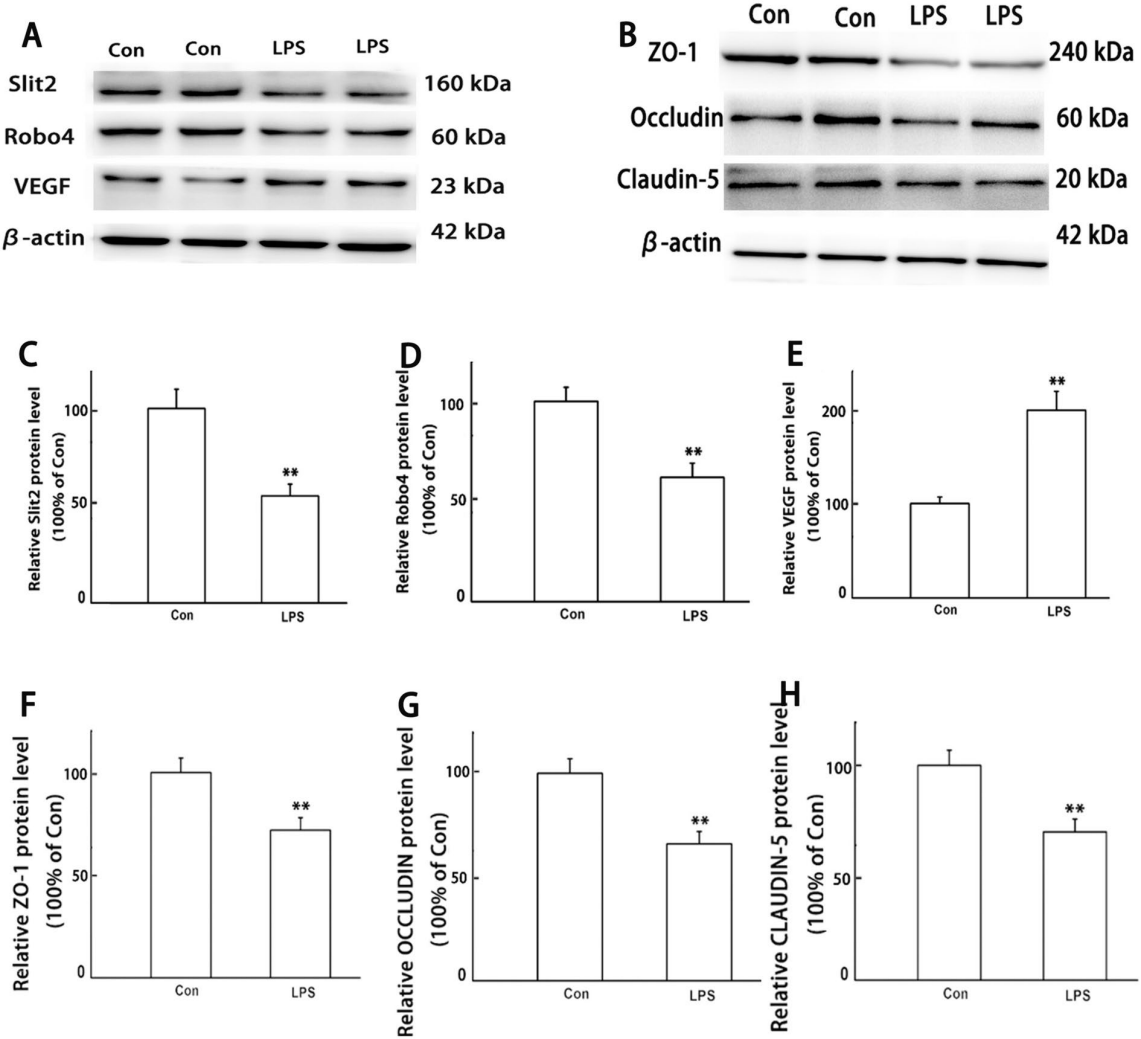
LPS-induced activation of Slit2–Robo4 signal pathway and disruption of tight junction. Slit2, Robo4, VEGF, ZO-1, Occludin, and Claudin-5 were detected by WB. All experiments were repeated twice. **A**, **B** Representative protein bands, C-H protein expression levels of Slit2, Robo4, VEGF, ZO-1, Occludin, and Claudin-5. Data are presented as the mean ± SEM, *n* = 8 in each group. ^**^P < 0.01 vs. control. *Con* control

Many literatures show that the occurrence of sepsis is related to the change of intestinal permeability [30, 31]. Therefore, we wonder to explore whether LPS induces inflammation in C57BL/6 J mice is associated with intestinal permeability or not. The immunohistochemistry and western blot results in Figs. 2 and 3 show that the intestine of LPS groups displayed decreased expression of Slit2, Robo4 and higher expression of VEGF when compared with Con group. Western blot results showed that LPS can induce decreased expression of tight junction protein ZO-1, Occludin, and Claudin-5.

### VX765 reversed LPS-induced inflammatory response mediated by NLRP3 inflammasome

**Fig. 4:**
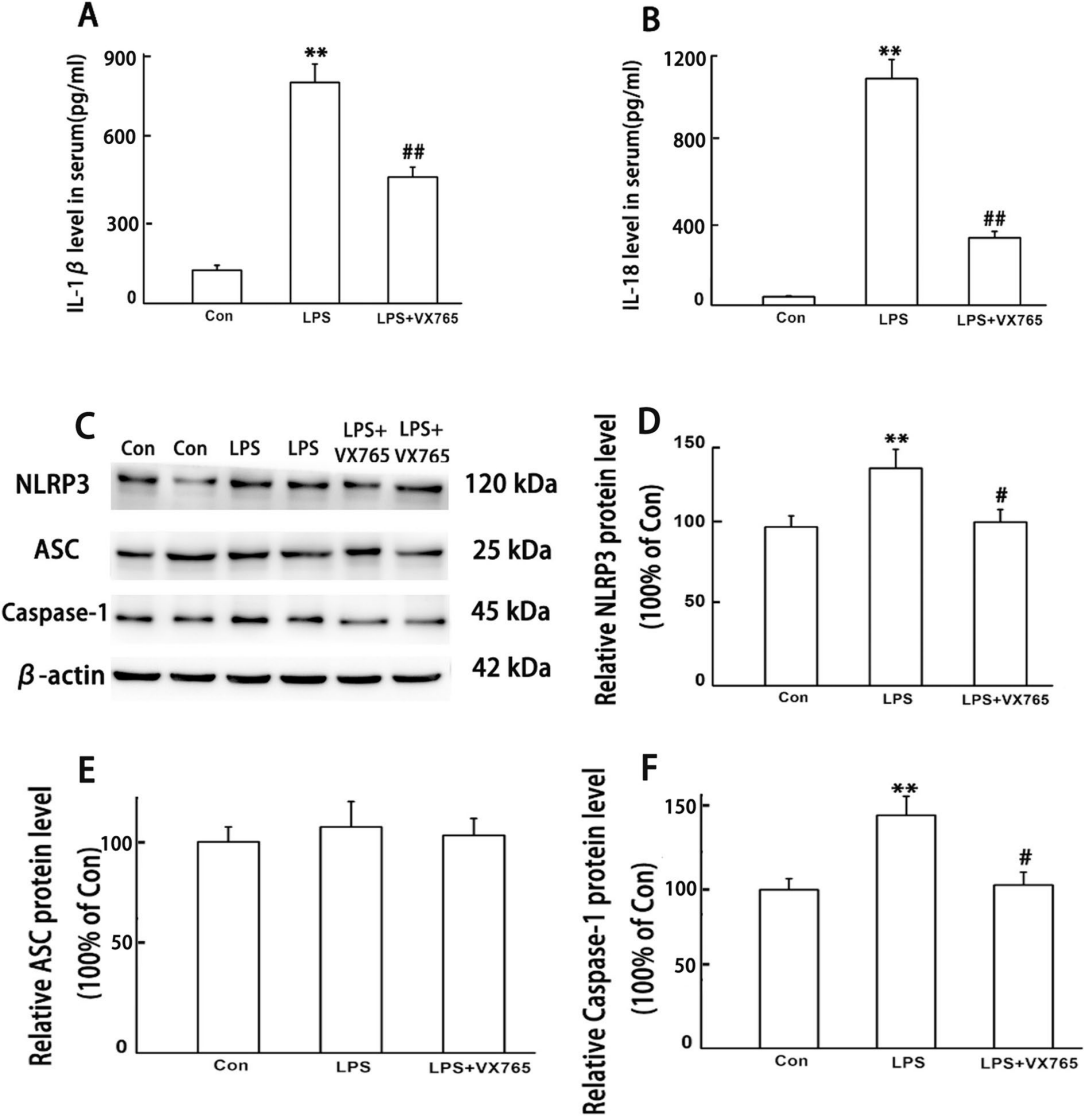
VX765 reversed LPS-induced inflammation and activation of NLRP3 inflammasome. IL-1β and IL-18 were detected by ELISA, NLRP3 inflammasome were detected by WB. All experiments were repeated twice. **A** IL-1β, **B** IL-18, **C** representative protein bands, **D** protein expression levels of NLRP3, **E** protein expression levels of ASC, **F** protein expression levels of Caspase-1. Data are presented as the mean ± SEM, *n* = 8 in each group. ^**^P < 0.01 vs. control; ^#^*P* < 0.05, ^##^*P* < 0.01 vs. LPS. *Con* control

In order to determine whether the increased IL-1β and IL-18 in serum induced by LPS injection is related to the activation of NLRP3 inflammasome, the second batch of experiments as described in Material and methods was carried out in this study. ELISA results showed that LPS-induced significant high level of IL-1β and IL-18 in serum can be reversed by VX765 when compared to LPS group (Fig. 4A, B). Western blot results showed that the expression of NLRP3 and Caspase-1 protein increased significantly after LPS injection for 24 h, which can also be reversed by VX765 (Fig. 4C–F).

### VX765 reversed the higher permeability of intestine and activation of Slit2–Robo4 signal pathway induced by LPS

**Fig. 5:**
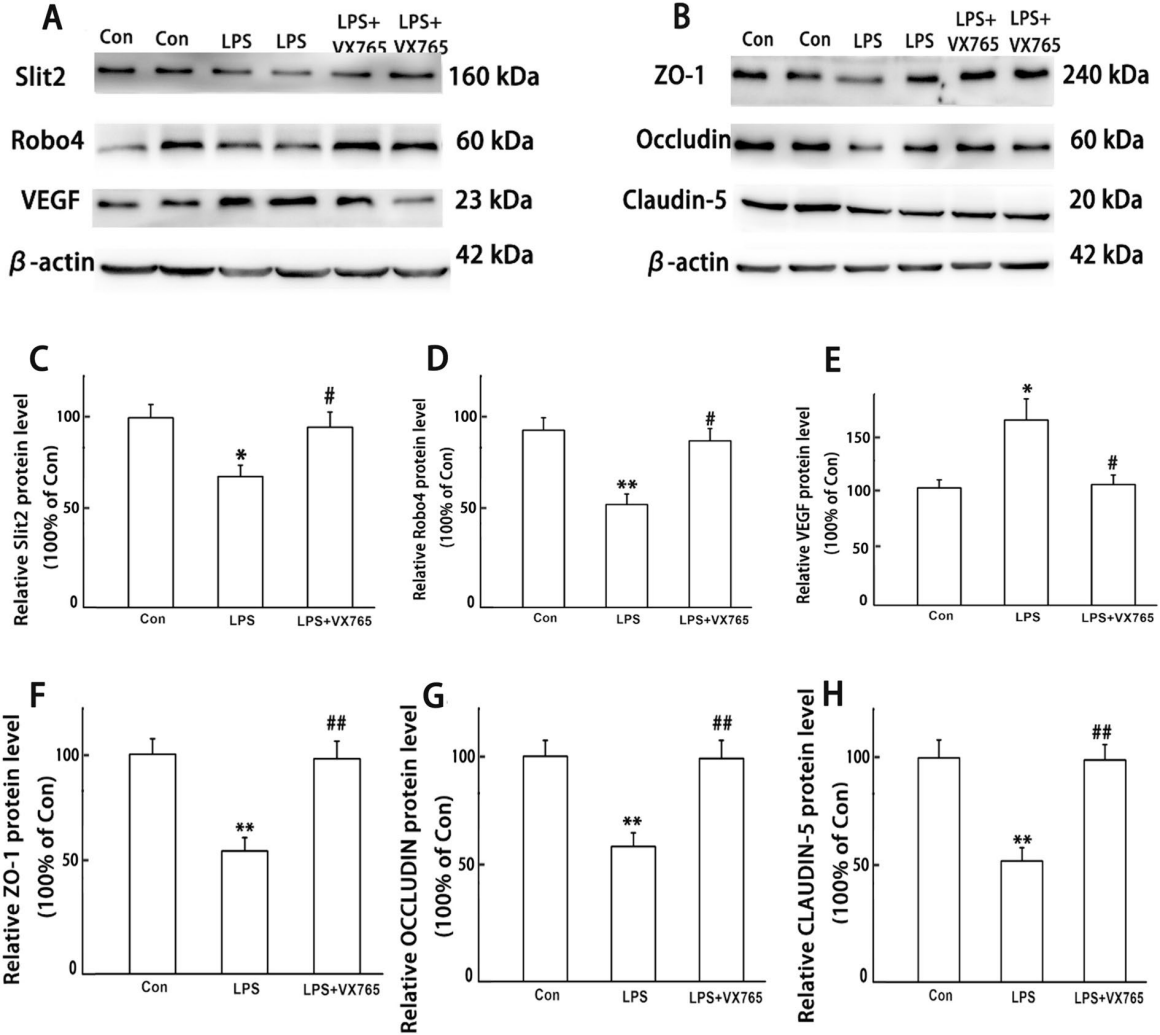
VX765 reversed LPS-induced activation of Slit2–Robo4 signal pathway and disruption of tight junction. Slit2, Robo4, VEGF, ZO-1, Occludin, and Claudin-5 were detected by WB. All experiments were repeated twice. **A**, **B** Representative protein bands, C-H protein expression levels of Slit2, Robo4, VEGF, ZO-1, Occludin, and Claudin-5. Data are presented as the mean ± SEM, *n* = 8 in each group. ^**^P < 0.01 vs. control; ^#^*P* < 0.05, ^##^*P* < 0.01 vs. LPS. *Con* control

In order to determine whether VX765 can reverse the permeability of intestine, western blot was used to examine the expression of Slit2, Robo4, VEGF, tight junction protein ZO-1, Occludin, and Claudin-5. The results in Fig. 5 showed that the intestine of LPS groups displayed decreased expression of Slit2, Robo4, tight junction protein ZO-1, Occludin, and Claudin-5 and higher expression of VEGF, all of which can be reversed by VX765 when compared to LPS group (Fig. 5).

## Discussion

Sepsis is a common, but poorly understood clinical syndrome with high motality in the world [32]. Although with the continuous progress of medical technology and updating of international guidelines, the death caused by sepsis is still increasing rapidly, which is because the pathogenesis has not been fully elucidated. Therefore, it is the common wish of researchers to elucidate the molecular mechanism of sepsis all over the world. In the present study, we showed that mice injected with LPS (5 mg/kg, i.p.) for 24 h could induce severe inflammatory reactions including an increased IL-1β, IL-18 in serum, activation of NLRP3 inflammasome, Slit2–Robo4 signaling pathway and disruption of tight junction in intestine. The injection of VX765 (10 mg/kg, i.p.), an inhibitor of NLRP3 inflammasome, could reverse these effects induced by LPS. Altogether, our findings revealed that Slit2–Robo4 signaling pathway and tight junction in intestinal probably involved in LPS-induced inflammation in mice, which may account for the molecular mechanism of sepsis.

A large number of researches have shown that barrier dysfunction and microvascular leakage of intestinal endothelial cells are the key factors of organ failure in sepsis and sepsis-related complications [33, 34]. The intestinal barrier consists of endothelial cells, adherens junctions, tight junctions, and extracellular components. Normally, the endothelial barrier is semi-permeable, which enables the transport of fluids and solutes from the blood to the tissues [35]. However, in patients with sepsis, abnormal barrier function leads to enhanced protein and solute transport. Breed et al. and other researchers have reported that the intestinal permeability of young C57BL/6 J female mice in cecal ligation and puncture group was higher than that of in the control group, which was related to the changes of claudins 1, 2, 3, 4, 5 and 8 [36, 37]. Wang et al. reported that the activation of autophagy and NLRP3 inflammasome by LPS + ATP can result in paracellular permeability increase and morphological disruption of both ZO-1 and Occludin [38]. In this study, tight junction protein ZO-1, Occludin, and Claudin-5 in the intestine of LPS group and Con group were examined by western blot. The results showed that LPS can induce decreased expression of tight junction protein ZO-1, Occludin, and Claudin-5. These effects can be reversed by VX765. All of these results demonstrated that inflammation induced by LPS is related to the increased permeability of intestine. However, this conclusion is preliminary, which also needs further experiments such as researches in gene knockdown animal model to prove it in the future.

Many studies have shown that endothelial cells specifically express receptor Robo4 which binding to its ligand Slit can inhibit inflammation and endothelial permeability by enhancing adhesion and adjusting cytoskeleton dynamics [39, 40]. Slit2 binds to the receptors Robo1 and Robo4, and then induces a series of intracellular signaling events. Slit2 regulates angiogenesis and protects endothelial integrity during sepsis and HIV infection [27]. In this study, Slit2 and Robo4 were examined by western blot in LPS group and Con group. The results showed that LPS can decrease the expression of Slit2 and Robo4, which is relative to the inflammation in sepsis. These effects also can be reversed by VX765. The mechanisms by which Slit2 regulates endothelial permeability are complex. The research results of Vincent et al. showed that Slit2N was the active fragment of Slit2, which could promote and accelerate the localization of VE cadherin and p120-catenin on the surface of endothelial cells [26, 41]. Therefore, Slit2N can reduce the permeability changes of endothelial cells induced by inflammatory mediators such as VEGF, LPS, TNF-α, and IL-1β [42, 43]. It was found that Slit2N reduced the accumulation of neutrophils and protein exudates in the alveolar cavity of LPS treated mice. But this effect is not present in Robo4 knockout mice, which proves the importance of this receptor [44]. In human umbilical vein endothelial cells (HUVECs), LPS downregulated Slit2, Robo4 and VE-cadherin protein expression and increased endothelial cell permeability in vitro during inflammation. Chen et, al. reported that hPMSC-derived Slit2 may inhibit LPS-induced CD11b, CD18 expression to decrease cell migration and increase adhesion through modulating the activity and motility of inflammatory macrophages in placenta [45, 46]. Vascular endothelial growth factor (VEGF), a kind of glycoprotein produced by endothelial cells, regulates vascular permeability through binding to VEGF receptors and activating the corresponding signal pathway [47]. Recently, some studies have focused on these effects of sepsis [48]. In this study, VEGF was examined, and the results showed that LPS can induce higher expression of VEGF which can be reversed by VX765. These results indicated that the increased permeability of the endothelium is associated with high expression of VEGF, but the underlying mechanisms need to be elucidated.

Although the experimental results of this paper are very interesting, we recognize the study still has some limitations. Firstly, this study did not carry out functional experiments to verify the established sepsis animal model, such as measuring heart rate, blood pressure and statistical mortality. Secondly, in the experimental design, the detection indexes that measured only after LPS injection for 24 h. The time point was single, and it would be more meaningful to examine the relevant detection indexes of animals after LPS injection at different time points, which can reflect the whole dynamic process of inflammatory factors with time. Third, there was no corresponding inhibitor and/or agonist group or used gene knockout technology to verify the Slit2–Robo4 signaling pathway. These deficiencies are interesting and worth for studying in the future by setting more experimental groups, time points and inflammatory factors and so on. What is more, Slit2 knockout mouse model could also be established, which can be used for further observing tight junction and other indicators by immunofluorescence or transmission electron microscope.

In conclusion, our study indicated that LPS could induce severe inflammatory reactions including an increased IL-1β, IL-18 in serum, activation of NLRP3 inflammasome, Slit2–Robo4 signal pathway and disruption of tight junction in intestine. VX765, an inhibitor of NLRP3 inflammasome, could reverse these effects induced by LPS. Altogether, our novel findings revealed that Slit2–Robo4 signaling pathway and tight junction in intestinal may be involved in LPS-induced inflammation in mice, which may account for the molecular mechanism of sepsis.

## Data Availability

All data generated or analyzed during this study are included in this published.

## Abbreviations

LPS: Lipopolysaccharide
IL-1β: Interleukin-1β
IL-18: Interleukin-18
TNF-α: Tumor necrosis factor-α
IFN-γ: Interferon-γ
NLRP3: Nucleotide-binding oligomerization domain‑like receptor protein 3
ASC: Apoptosis‑associated speck‑like protein
VEGF: Vascular endothelial growth factor
